# Monoassociation with Lactobacillus plantarum Disrupts Intestinal Homeostasis in Adult Drosophila melanogaster

**DOI:** 10.1128/mBio.01114-18

**Published:** 2018-07-31

**Authors:** David Fast, Aashna Duggal, Edan Foley

**Affiliations:** a Department of Medical Microbiology and Immunology, University of Alberta, Edmonton, Alberta, Canada; Max Planck Institute for Marine Microbiology

**Keywords:** *Drosophila*, *Lactobacillus*, epithelial cells, host response, stem cells, symbionts

## Abstract

Adult *Drosophila melanogaster* raised in the absence of symbiotic bacteria have fewer intestinal stem cell divisions and a longer life span than their conventionally reared counterparts. However, we do not know if increased stem cell divisions are essential for symbiont-dependent regulation of longevity. To determine if individual symbionts cause aging-dependent death in *Drosophila*, we examined the impacts of common symbionts on host longevity. We found that monoassociation of adult *Drosophila* with Lactobacillus plantarum, a widely reported fly symbiont and member of the probiotic *Lactobacillus* genus, curtails adult longevity relative to germfree counterparts. The effects of Lactobacillus plantarum on life span were independent of intestinal aging. Instead, we found that association with Lactobacillus plantarum causes an extensive intestinal pathology within the host, characterized by loss of stem cells, impaired epithelial renewal, and a gradual erosion of epithelial ultrastructure. Our study uncovers an unknown aspect of Lactobacillus plantarum-*Drosophila* interactions and establishes a simple model to characterize symbiont-dependent disruption of intestinal homeostasis.

## IMPORTANCE

Under homeostatic conditions, gut bacteria provide molecular signals that support the organization and function of the host intestine. Sudden shifts in the composition or distribution of gut bacterial communities impact host receipt of bacterial cues and disrupt tightly regulated homeostatic networks. We used the Drosophila melanogaster model to determine the effects of prominent fly symbionts on host longevity and intestinal homeostasis. We found that monoassociation with Lactobacillus plantarum leads to a loss of intestinal progenitor cells, impaired epithelial renewal, and disruption of gut architecture as flies age. These observations uncover a novel phenotype caused by monoassociation of a germfree host with a common symbiont and establish a simple model to characterize symbiont-dependent loss of intestinal homeostasis.

## INTRODUCTION

Environmental, microbial, and host factors establish an intestinal environment that permits colonization by a variable consortium of bacteria. Extrinsic factors such as pH, oxygen, and nutrient supply influence the biogeography of microbe distribution, while physical barriers contain microbes within the gut lumen. Host-derived bacteriostatic products such as antimicrobial peptides and reactive oxygen species (ROS) limit bacterial numbers and prevent invasion of the host interior. Inside the lumen, microbes compete with each other for access to nutrients and intestinal attachment sites and release metabolites that influence host processes as diverse as growth, immunity, and behavior (1–6). Shifts in the composition or distribution of this bacterial community often lead to the onset of debilitating, and potentially deadly, diseases for the hosts (4, 7–9).

Drosophila melanogaster is a useful model to study interactions between a host and individual species of symbiotic bacteria (10). The fly microbiome consists of a limited number of bacterial species that are easily cultured and manipulated in isolation (11–14). Researchers have access to simple protocols for the establishment of gnotobiotic fly cultures (15), and flies lend themselves to sophisticated manipulation of host gene expression. Of equal importance, there are extensive genetic, developmental, and biochemical similarities between fly and mammalian gut biology (16–19). Thus, discoveries in *Drosophila* provide insights into evolutionarily conserved features of host-bacterium interactions. For example, in flies and mammals, basal intestinal stem cells (ISCs) divide and differentiate at a rate that maintains an intact epithelial barrier (17, 20, 21). A relatively simple “escalator” program times ISC division to match the loss of aged cells, while a more complex, adaptive program activates ISC division to compensate for environmental destruction of host cells (22–26). This adaptive regulation of growth maintains the integrity of the epithelial barrier and is critical for long-term health of the host. Breaches to the gut barrier permit an invasion by intestinal microbes that activate local immune responses and drive the development of chronic inflammatory illnesses (4, 9, 27, 28).

Although the microbiome of *Drosophila* is orders of magnitude less complex than that found in mammals (12), populations of *Lactobacillus* species are common to fly midguts and animal small intestines (11–13). Studies of the *Drosophila* symbiont Lactobacillus plantarum (L. plantarum) uncovered several interactions between the two species. *L. plantarum* contributes to larval growth (29), uptake of dietary protein (30), and management of malnutrition in the host (31). Furthermore, *L. plantarum* induces ROS generation by NADPH oxidase (32) and protects the flies from damaging agents (33). Remarkably, many host responses to *L. plantarum* are conserved across large evolutionary distances, as *L. plantarum* strains also coordinate nutrient acquisition (31), ROS generation (32), and growth and gut defenses in the mouse (31, 33). These observations position the fly as a valuable model to examine developmental and homeostatic contributions of *Lactobacillus* to animal health (34).

Our interest in *L. plantarum* arose from previous data indicating that elimination of the *Drosophila* microbiome slows ISC turnover and extends adult longevity (9, 23, 35). These observations led us to ask if symbiotic bacteria reverse the germfree (GF)-mediated extension of fly life span by accelerating the division of ISCs. To test this hypothesis, we examined the effects of common fly symbionts on GF host longevity. Of all species tested, we found that *L. plantarum* recapitulated the microbiome-mediated truncation of GF adult life span. However, counter to our initial expectation, we did not find that *L. plantarum* increased ISC division rates. Instead, we found that monoassociation of adult flies with *L. plantarum* led to a loss of ISCs, a block to ISC renewal, and a gradual deterioration of epithelial integrity upon aging. Combined, our data show that long-term monoassociation of adult *Drosophila* with *L. plantarum* destabilizes the intestine and shortens host longevity.

(This article was submitted to an online preprint archive [36].)

## RESULTS

### Lactobacillus plantarum outcompetes Lactobacillus brevis for association with adult *Drosophila*.

**FIG 1:**
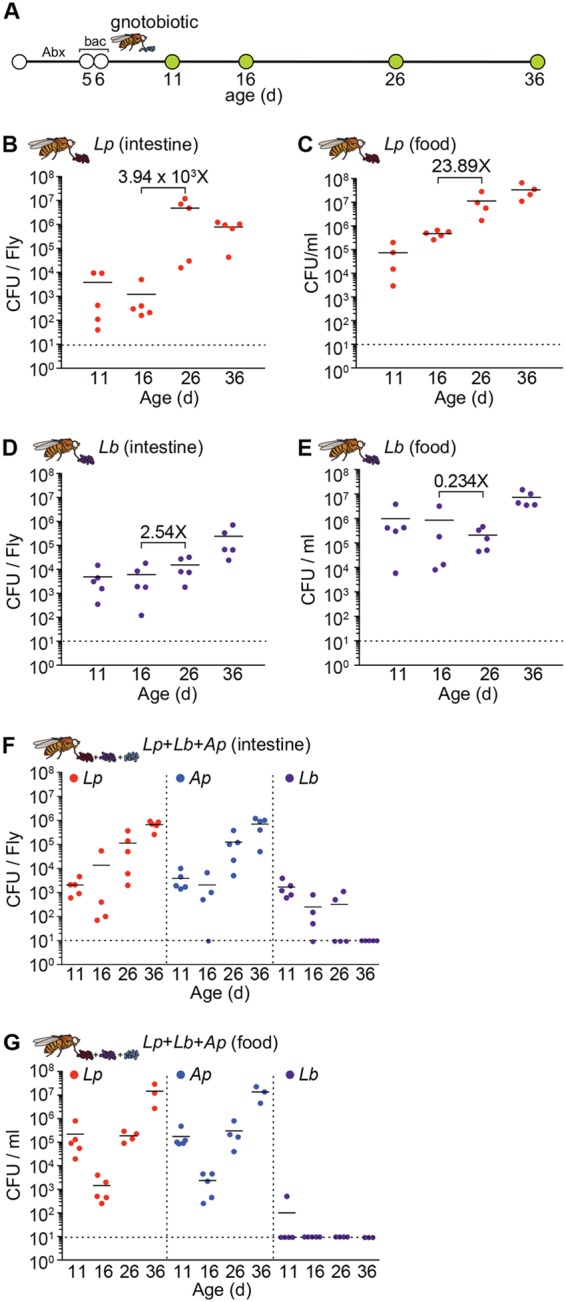
*L. plantarum* outcompetes *L. brevis* in the adult gut. (A) Schematic representation of the experimental timeline and generation of gnotobiotic adult flies. “Abx” indicates duration of antibiotic treatment, and “bac” indicates duration of bacterial feeding. (B to E) CFU per fly of *L. plantarum* (Lp) and *L. brevis* (Lb) in the intestines (B and D) and on the food (C and E) of *L. plantarum*-monoassociated and *L. brevis*-monoassociated adult flies, respectively, at days 11, 16, 26, and 36 of age. (F and G) CFU per fly of *L. plantarum*, *L. brevis*, and *A. pasteurianus* (Ap) in the intestines (F) and on the food (G) of *L. plantarum*-*L. brevis*-*A. pasteurianus*-polyassociated adult flies. Black numbers on graphs denote fold change in the mean between indicated time points.

Our lab strains of *Drosophila* predominantly associate with L. plantarum, Lactobacillus brevis (L. brevis), and Acetobacter pasteurianus (A. pasteurianus) (37). Of those strains, lactobacilli, particularly *L. plantarum*, are the dominant symbionts, typically accounting for >75% of all bacterial operational taxonomic units (OTUs) in flies that we raise on standard cornmeal medium. As fly symbionts regularly cycle from the intestine to the food (38–40), we conducted a longitudinal study of the association of *L. plantarum* and *L. brevis* with cultures of virgin female wild-type *Drosophila*. For this work, we fed freshly emerged adult flies an antibiotic cocktail to eliminate the endogenous bacterial microbiome (35, 41). We then fed antibiotic-treated adult flies equal doses of *L. plantarum* or *L. brevis* for 16 h, transferred flies to fresh food, and determined bacterial titers in the intestine and food at regular intervals thereafter (Fig. 1A).

We typically found less than 1 × 10^4^ CFU per fly gut 5 days after inoculation with either *L. plantarum* or *L. brevis* (Fig. 1B and D). In both cases, intestinal bacterial loads increased over time. However, the effect was more pronounced for *L. plantarum* than *L. brevis*. We detected a mean 4 × 10^4^-fold increase in numbers of *L. plantarum* associated with the fly gut between days 16 and 26, rising to approximately 1 × 10^7^ CFU per fly gut by day 26. In contrast, we observed only a 2.5-fold increase in *L. brevis* gut association over the same time, yielding less than 1 × 10^5^ CFU per fly gut. Likewise, we found that the *L. plantarum* load steadily increased in the food over time (Fig. 1C), while the association of *L. brevis* with food remained relatively constant (Fig. 1E). These observations suggest that *L. plantarum* has a growth advantage over *L. brevis* when cocultured on fly food with adult *Drosophila*. To determine if *L. plantarum* outcompetes *L. brevis* for association with *Drosophila*, we fed germfree adult flies a 1:1:1 mixed culture of *L. plantarum*, *L. brevis*, and *A. pasteurianus* and monitored bacterial association rates over time. We added *A. pasteurianus* to the culture in this experiment to more accurately represent the microbiome of our conventional lab flies. Of this defined bacterial community, we found that *L. plantarum* and *A. pasteurianus* populated the fly intestine (Fig. 1F) and food (Fig. 1G) with near-equal efficiency. In both cases, the microbial load associated with the gut or food increased over time, typically reaching approximately 1 × 10^6^ CFU per intestine 36 days after inoculation. Intestinal association by *L. plantarum* was an order of magnitude higher in monoassociated flies (Fig. 1B) than in polyassociated flies (Fig. 1F), suggesting that *A. pasteurianus* partially limits host association with *L. plantarum*. In contrast to *L. plantarum* and *A. pasteurianus*, we found that *L. brevis* gradually disappeared from the food and the intestines of polyassociated adult flies over time (Fig. 1F and G). By 36 days, we repeatedly failed to detect *L. brevis* in the intestine or food. Combined, these observations suggest that the *L. plantarum* and *A. pasteurianus* strains used in this study are more effective at forming persistent, long-term associations with *Drosophila* than the *L. brevis* strain and may explain the predominance of *L. plantarum* and *A. pasteurianus* in fly cultures.

### Host genetic background influences transcriptional responses to intestinal microbes.

As *L. plantarum* and *A. pasteurianus* form long-term associations with adult *Drosophila*, we tested the effects of the respective strains on adult longevity. This experiment requires the generation of GF flies that we subsequently associate with defined bacterial cultures. For the data in Fig. 1, we generated GF adult flies by supplementing the food with antibiotics. In an alternative method, investigators incubate embryos in a bleach solution that removes all associated microbes and establishes an axenic organism that develops in the absence of symbiotic bacteria (15). To determine if the respective methods have distinct impacts on transcription in the gut, we compared microarray data on microbe-dependent gene expression in GF flies derived from bleached embryos or from antibiotic-treated adults. Specifically, we compared microbe-dependent transcriptional changes in the intestines of Oregon R and Canton S flies derived from bleached embryos (42) to microbial responses in the intestines of *w*; *esgGAL4, GAL80*^*ts*^*, UAS-GFP (esg*^*ts*^*)* antibiotic-treated adults (43). The *esg*^*ts*^ genotype is a variant of the *Drosophila* TARGET system (44) and is commonly used for temperature-dependent expression of upstream activation sequence (UAS)-bearing transgenes in green fluorescent protein (GFP)-marked ISCs and enteroblasts, collectively referred to as progenitor cells (23, 45). For this study, we used PANTHER to identify gene ontology (GO) terms that were significantly enriched in conventionally reared (CR) fly intestines relative to GF intestines for all three fly lines.

**FIG 2:**
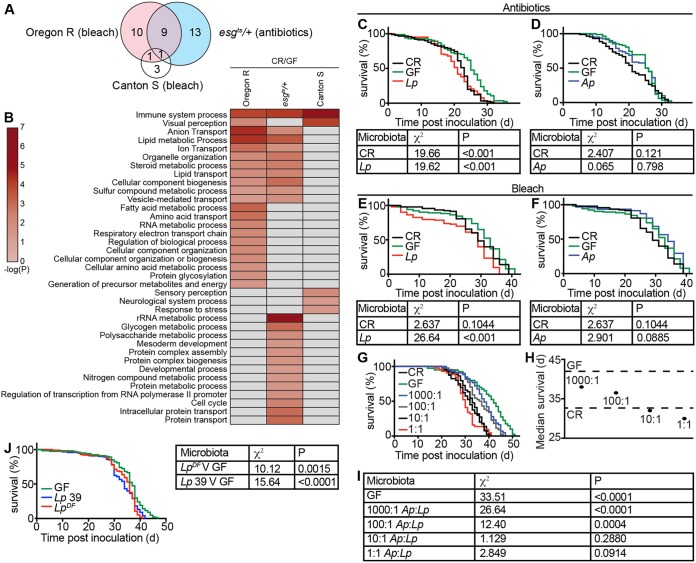
Monoassociation with symbiotic *L. plantarum* reduces GF adult fly life span. (A and B) Microbe-dependent gene expression microarray data from the intestines of Oregon R and Canton S flies from bleached embryos (42) and from the intestines of *esg*^*ts*^ antibiotic-treated adults (43). The heat map (B) shows gene ontology terms that were significantly enriched in the respective groups, and the Venn diagram (A) shows overlapping gene ontology terms between each group. (C) Survival curve of CR, GF, and *L. plantarum*-monoassociated adult flies from GF adults generated with antibiotics. (D) Survival curve of CR, GF, and *A. pasteurianus*-monoassociated adult flies from GF adults generated with antibiotics. (E) Survival curve of CR, GF, and *L. plantarum*-monoassociated adult flies generated from bleached embryos. (F) Survival curve of CR, GF, and *A. pasteurianus*-monoassociated adult flies generated from bleached embryos. (G) Survival curves of CR and GF flies and flies coassociated with *A. pasteurianus/L. plantarum* at indicated ratios. For each graph (C to G and J), the *y* axis represents percent survival and the *x* axis represents time post-bacterial inoculation. (H) Median survival from data represented in panel G. Dashed lines show median survival times for GF and CR flies. (I) Comparisons of survival data for the indicated treatment groups relative to CR flies. (J) Survival curve of monoassociated adult flies associated with one of two different strains of *L. plantarum* (*L. plantarum* DF or *L. plantarum* 39) and comparisons of survival data for GF flies versus flies associated with the indicated *L. plantarum* strains. All χ^2^ and *P* values are relative to GF flies. Tables are results of log rank (Mantel-Cox) test for panel data.

In this comparison, we did not see clear distinctions between the effects of bleach and antibiotics on transcriptional outputs from the gut (Fig. 2A and B). In each case, removal of the microbiome altered the expression of immune response genes (Fig. 2B), a result that matches earlier data linking gut bacteria and intestinal immunity (42).

Further analysis suggested that changes in microbe-dependent gene expression were influenced to a greater extent by fly genotype rather than by the method used to ablate the microbiome. For example, of the remaining microbe-responsive GO terms, we noticed a more pronounced similarity between bleached Oregon R flies and antibiotic-treated *esg*^*ts*^ flies. Of the 21 processes affected by bleach treatment of Oregon R cultures, 10 were similarly affected by antibiotic treatment of *esg*^*ts*^ flies (Fig. 2A and B). In contrast, removal of the microbiome with bleach had a mild effect on gut transcription in Canton S flies. In this case, bleach affected only five GO terms, three of which are unique to Canton S (Fig. 2A and B). As a caveat to these interpretations, we note that uncontrolled variables such as differences between the microbiomes of the respective CR flies may impact the differences noted in these comparisons. Nonetheless, these results suggest that host genetic background contributes to the effects of the microbiome on intestinal gene expression.

### Monoassociation with L. plantarum shortens adult longevity relative to germfree counterparts.

We then asked if the method of bacterial elimination influences host survival after reassociation with symbiotic bacteria. For this assay, we prepared GF adults from bleached eggs or from CR adults raised on antibiotic-treated food and measured the longevity of flies associated with one of two common fly symbionts. Specifically, we inoculated the respective GF adult flies with *A. pasteurianus* or *L. plantarum* and measured their life spans relative to CR counterparts. Irrespective of the means used to generate GF flies, we found that *L. plantarum* significantly shortened the life span of adult *Drosophila* (Fig. 2C and E). These observations match recent reports that GF adults outlive flies monoassociated with additional *L. plantarum* strains (46, 47). In addition, we found that flies associated with either the *L. plantarum* DF strain isolated from a wild Drosophila melanogaster fly (37) or the *L. plantarum* 39 strain, isolated from pickled cabbage (48), also have shorter life spans than GF controls (Fig. 2J). Combined, these data indicate that monoassociation of adults with *L. plantarum* reverses the life span extension noted in GF flies. In contrast, monoassociation of adult *Drosophila* with *A. pasteurianus* had no effect on adult life span, regardless of the method used to generate GF flies (Fig. 2D and F). As *A. pasteurianus* attenuates gut colonization by *L. plantarum* (Fig. 1F) and *A. pasteurianus* does not affect adult life span, we tested if *A. pasteurianus* attenuates the impacts of *L. plantarum* on GF life span extension. For these assays, we measured the life spans of GF adults that we cultured with different ratios of *A. pasteurianus* and *L. plantarum*. Here, we observed a clear relationship between *A. pasteurianus*/*L. plantarum* input ratios and adult life span—the greater the ratio of *A. pasteurianus* to *L. plantarum*, the longer the life span of coassociated flies (Fig. 2G to I). Together, these data argue that monoassociation with *L. plantarum* reverts the life span extension observed in GF flies.

### *L. plantarum* does not activate proliferative responses in the host intestine.

**FIG 3:**
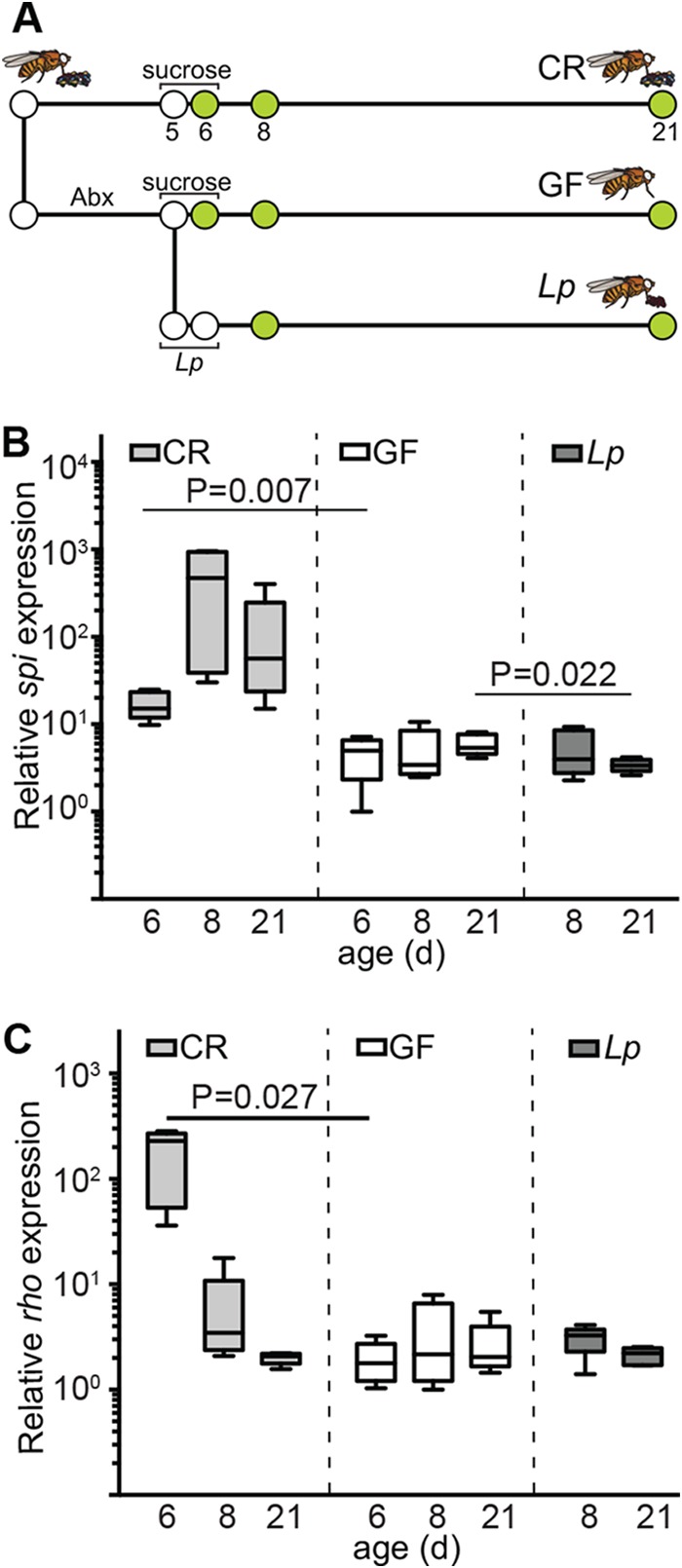
*L. plantarum* does not trigger a proliferative response in adult fly intestines. (A) Schematic representation of gnotobiotic fly generation and experimental timeline. “Abx” indicates duration of antibiotic treatment. Sucrose and *L. plantarum* show feeding regimes for the respective groups. Green circles indicate times at which samples were processed. (B and C) Quantitative real-time PCR analysis of expression of the EGF-type growth factor *spitz* (B) and the *spitz*-activating endopeptidase rhomboid (C) from the dissected guts of adult CR, GF, and *L. plantarum*-associated flies. Each time point represents five independent measurements. *P* values are the results of pairwise comparisons from a one-way ANOVA.

In *Drosophila*, symbiotic bacteria provide mitogenic cues that accelerate the growth and aging of intestinal tissues (23), a factor associated with host longevity (49). This prompted us to test if *L. plantarum* activates ISC division. Initially, we quantified expression of the epidermal growth factor (EGF) ligand *spitz* and the *spitz*-activating endopeptidase *rhomboid* in dissected intestines. We selected the EGF pathway for this study as EGF activates ISC proliferation in response to symbiotic bacteria (23) and damage to the intestinal epithelium (22). Consistent with a relationship between gut bacteria and ISC proliferation, we detected significantly higher levels of *spitz* (Fig. 3B) and *rhomboid* (Fig. 3C) in CR flies than in GF flies. In contrast, we did not observe expression of EGF pathway activators in the intestines of flies associated with *L. plantarum* (Fig. 3B and C). Instead, we found that *spi* was expressed at significantly lower levels in the midguts of *L. plantarum*-monoassociated flies than in GF flies 15 days after association (Fig. 3B). These data suggest that monocolonization of the adult intestine with *L. plantarum* fails to activate EGF-dependent proliferative responses in the host intestine.

### Impaired epithelial renewal in *L. plantarum*-monoassociated flies.

**FIG 4:**
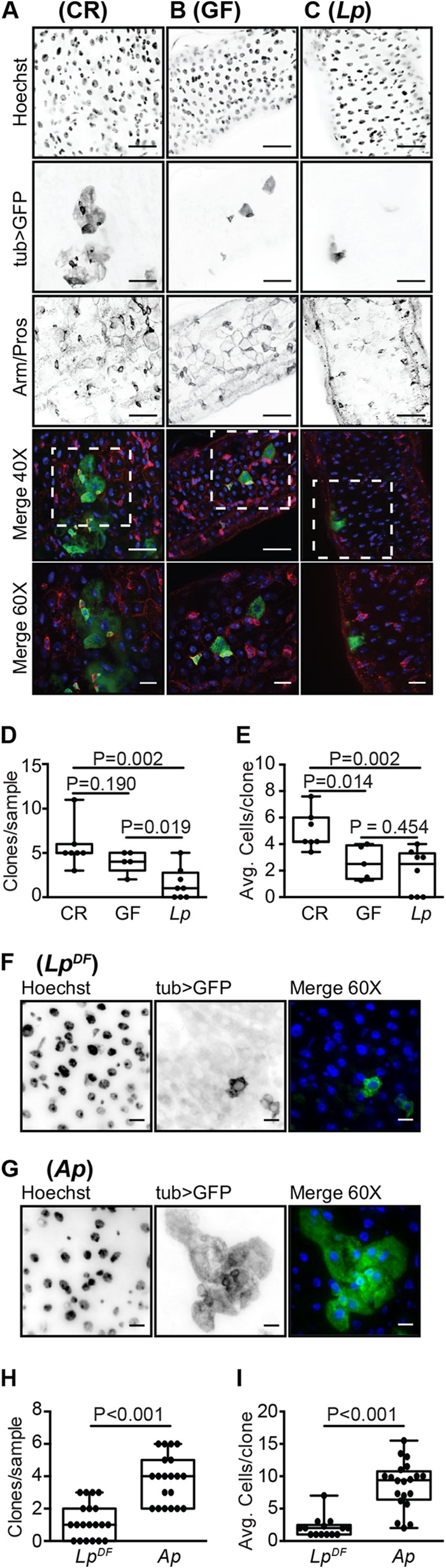
A lack of epithelial renewal in the guts of *L. plantarum*-monoassociated flies. (A to C) GFP-positive MARCM clones from the posterior midgut of CR (A), GF (B), and *L. plantarum*-monoassociated (C) flies at day 26 of age. Guts were stained with Hoechst stain and anti-Armadillo/Prospero antibodies as indicated. Hoechst stain (blue), GFP (green), and Armadillo/Prospero (red) were merged in the fourth (×40 magnification) and fifth rows. Boxed regions in the fourth row are shown at a higher magnification (×60) in the fifth row. (D and E) Quantification of clones per sample (D) and cells per clone (E) in CR, GF, and *L. plantarum*-monoassociated flies. (F and G) GFP-positive MARCM clones from the posterior midgut of *L. plantarum* DF-monoassociated (F) or *A. pasteurianus*-monoassociated (G) flies at day 26 of age. Guts were stained with Hoechst stain. Hoechst stain (blue) and GFP (green) were merged in the third column (×60). (H and I) Quantification of clones per sample (H) and cells per clone (I) in *L. plantarum* DF- and *A. pasteurianus*-monoassociated flies. For all images, ×40 bars are 25 µm and ×60 bars are 10 µm. *P* values are the results of pairwise comparisons from a one-way ANOVA.

To more accurately determine the effects of *L. plantarum* on ISC proliferation, we used the MARCM clonal marking method to assess stem cell proliferation in the intestines of CR, GF, and *L. plantarum*-associated flies. MARCM labels all progeny of an ISC division with GFP (50). As a result, clone number and size provide a simple proxy for total divisions in the midgut. We looked at ISC division in CR flies, GF flies, and flies that we associated with *L. plantarum*. In each case, we counted the total number of mitotic clones per posterior midgut and the number of cells per clone. As expected, we noticed greater mitotic activity in the intestines of CR flies than GF flies. CR flies had significantly more mitotic clones than GF counterparts (Fig. 4A, B, and D), and CR clones contained significantly more cells than GF clones (Fig. 4E). In contrast to CR flies, monoassociation with *L. plantarum* failed to initiate proliferative responses in the host (Fig. 4C). In fact, the midgut contained significantly fewer clones than CR flies, or GF flies (Fig. 4D), and the clones that we observed in *L. plantarum*-associated flies invariably had fewer cells than age-matched clones in CR flies (Fig. 4E). To determine if impaired epithelial renewal occurs upon monoassociation with different strains of *L. plantarum*, we assessed stem cell proliferation in the intestines of flies that we monoassociated with *L. plantarum* DF. We noticed a similar absence of epithelial renewal in flies that we monoassociated with the *L. plantarum* DF strain, suggesting that this phenotype is not limited to a single strain of *L. plantarum* (Fig. 4F). In contrast, we observed significant levels of epithelial growth in the intestines of adult flies that we associated with *A. pasteurianus* (Fig. 4G to I), confirming that GF flies are not impaired in their ability to renew the intestinal epithelium upon reassociation with symbionts. These results, in conjunction with our quantitative measurements of host gene expression (Fig. 3), demonstrate a near-complete absence of epithelial renewal in intestines associated exclusively with *L. plantarum*.

### *L. plantarum*-monoassociated flies lack intestinal progenitors.

**FIG 5:**
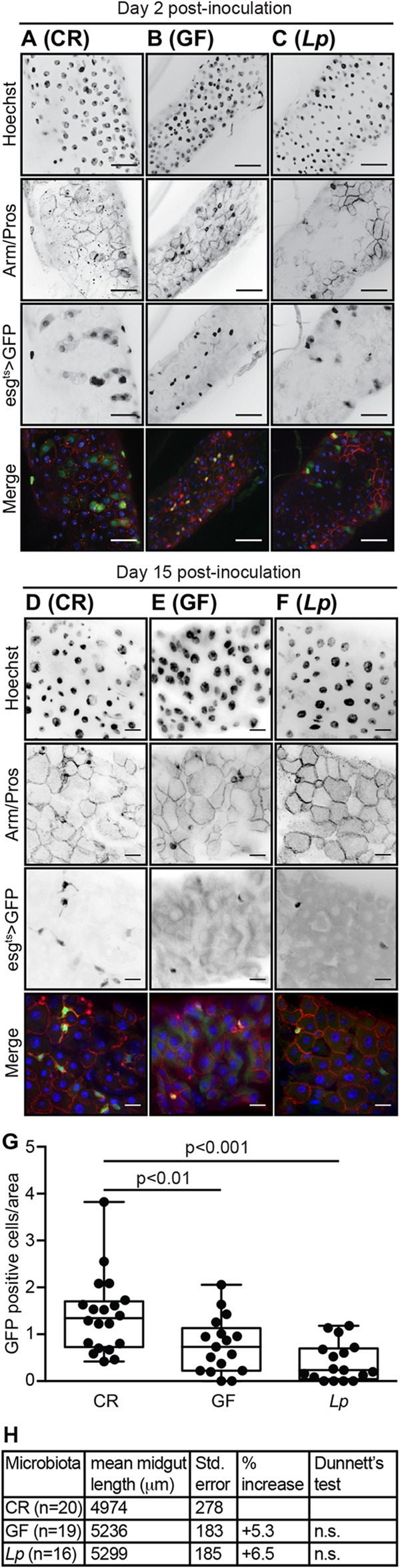
*L. plantarum*-monoassociated fly guts have low numbers of intestinal progenitor cells. (A to C) Immunofluorescence of posterior midguts of CR (A), GF (B), and *L. plantarum*-monoassociated (C) flies at day 11 of age. Bars, 25 µm. (D to F) Immunofluorescence of posterior midguts of CR (D), GF (E), and *L. plantarum*-monoassociated (F) flies at day 26 of age. Bars, 10 µm. Guts were stained with Hoechst stain and anti-Armadillo/Prospero antibodies as indicated. Progenitor cells were visualized with GFP as indicated. Hoechst stain (blue), GFP (green), and anti-Armadillo/Prospero (red) were merged in the fourth and eighth rows. (G) Quantification of progenitor numbers per unit surface area at day 26 of age. *P* values are the results of pairwise comparisons from a one-way ANOVA. (H) Mean midgut length of CR, GF, and *L. plantarum*-monoassociated flies at day 15 postinoculation. n.s., not significant.

Given the absence of ISC proliferation, we used immunofluorescence to determine if prolonged monoassociation with *L. plantarum* affected the cellular organization of posterior midguts. To measure the influence of *L. plantarum* on midgut morphology, we visualized the posterior midguts of CR, GF, and *L. plantarum*-monoassociated *esg*^*ts*^ flies that we raised for 2 or 15 days. We used GFP fluorescence and anti-Armadillo and anti-Prospero immunofluorescence to visualize progenitor cells (ISCs and enteroblasts), cell borders, and enteroendocrine cells, respectively. We did not observe differences between the different treatment groups at the early time point (Fig. 5A to C). In each case, midguts displayed the hallmarks of young intestines—evenly spaced nuclei, regular arrangements of GFP-positive progenitors, and neatly organized cell boundaries. As expected, 15 days postinoculation, CR midguts showed signs of age-dependent dysplasia (Fig. 5D). We no longer observed regular spacing between individual nuclei, Prospero and Armadillo stains revealed a disorganized epithelium, and the population of GFP-positive progenitors had expanded relative to 2-day-old fly guts. Consistent with bacterial contributions to the aging of the host intestine, we did not see a similar degree of dysplasia in GF flies. GF flies had regularly spaced nuclei, an organized epithelium, and fewer GFP-positive progenitor cells (Fig. 5E). We also saw minimal signs of dysplasia in the intestines of flies that we associated with *L. plantarum* for 15 days. In this case, we observed regularly spaced nuclei, defined cell borders, and an even distribution of enteroendocrine cells at day 15 (Fig. 5F). However, we noticed that *L. plantarum*-associated guts had approximately half the progenitor number of GF guts and significantly fewer progenitors than CR guts (Fig. 5G). We then examined the impacts of *L. plantarum* on the length of adult posterior midguts as microbial association affects midgut length in adult *Drosophila* (42, 51). Consistent with an earlier report (42), we noticed a similar, albeit milder, effect of microbial removal on the length of the adult intestine. On average, we found that the intestines of GF flies were 5% longer than CR controls (Fig. 5H). Similarly, we found that the intestines associated with *L. plantarum* were, on average, 6% longer than CR controls (Fig. 5H). However, we did not detect a statistically significant difference in mean gut length between the three treatments. We speculate that the relatively mild effects of bacterial removal on intestinal length noted in our study may be the result of fly strain differences or differences in fly culture methods or may occur as a result of removing the microbiome after completion of juvenile development. Nonetheless, our data suggest a detrimental impact of *L. plantarum* monoassociation on the pool of progenitor cells in adult flies.

### *L. plantarum* disrupts posterior midgut ultrastructure.

**FIG 6:**
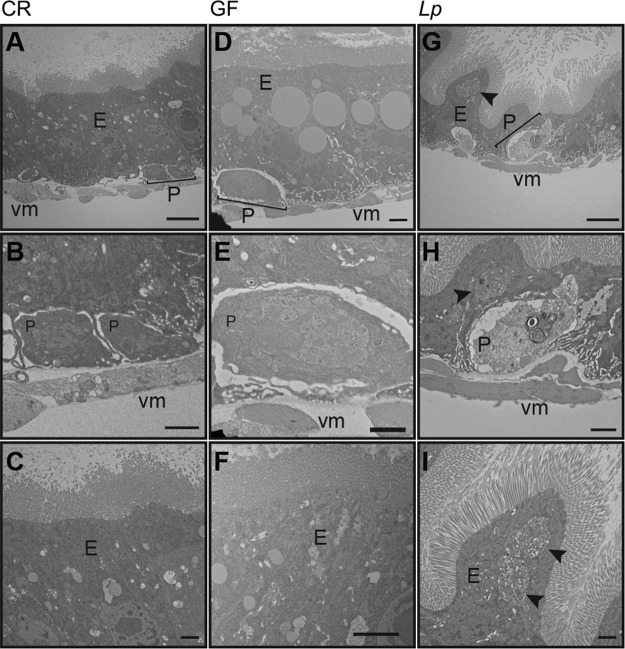
*L. plantarum* disrupts posterior midgut ultrastructure. Transmission electron microscopy of CR (A to C), GF (D to F), and *L. plantarum*-monoassociated (G to I) fly posterior midguts 15 days after inoculation. Epithelium (E), progenitors (P), and visceral muscle (vm) are lableled. Arrowheads indicate large vacuoles. (A, D, and G) Direct magnification, ×1,200. Bars, 5 µm. (B, E, and H) Direct magnification, ×3,000. Bars, 1 µm. (C, F, and I) Direct magnification, ×3,500. Bars, 1 µm.

As monoassociation with *L. plantarum* results in a loss of intestinal progenitors and a failure of epithelial renewal, we used transmission electron microscopy (TEM) to directly examine the effects of 15 days of monoassociation with *L. plantarum* on posterior midgut ultrastructure. As controls, we visualized the posterior midguts of age-matched CR and GF flies. CR midguts had the anticipated sheath of visceral muscle that surrounds small, basal cells, and large, columnar epithelial cells (Fig. 6A to C). As it is not possible to distinguish between ISCs and enteroblasts with TEM of this kind, we refer to the small basal cells as progenitor cells. In many ways, GF flies mirrored CR flies, with an organized visceral musculature (Fig. 6D), basal progenitors (Fig. 6D and E), and an intact brush border (Fig. 6F). Upon examination of midguts associated with *L. plantarum*, we were struck by substantial alterations to intestinal morphology. The epithelium contained an undulating population of cells (Fig. 6G and H) with large vacuoles (Fig. 6G to I, arrowheads) and poorly discernible nuclei (Fig. 6G). We also noticed alterations to the morphology of presumptive progenitor cells. In place of the small, densely stained progenitors intimately associated with the visceral muscle of CR or GF flies, monoassociation with *L. plantarum* resulted in the appearance of misshapen cells that did not associate properly with the muscle and had large, lightly stained nuclei and numerous cytosolic vacuoles (Fig. 6G and H). These findings show that monocolonization of a GF adult midgut with *L. plantarum* causes an intestinal phenotype that is characterized by thinning of the epithelium, formation of large cytosolic vacuoles, and a loss of progenitor cells. In summary, monoassociation of adult *Drosophila* with *L. plantarum* results in an intestinal phenotype that is distinct from CR or GF flies. *L. plantarum* forms a persistent association with GF *Drosophila* that impairs epithelial renewal programs, depletes progenitor cell populations, and ultimately shortens host longevity.

## DISCUSSION

Gut bacteria activate homeostatic ISC division programs that are critical for the maintenance of a healthy digestive tract (23). Failure to regulate stem cell division exposes the host to microbial invasion and potentiates the development of chronic inflammatory illnesses (23, 27). In this report, we used the *Drosophila* model to examine the effects of symbiotic bacteria on adult longevity and execution of the epithelial renewal program. We asked how *L. plantarum* affects adult longevity and ISC division, as recent studies suggest that interruption of stem cell division does not have substantial effects on life span (35, 52). We found that monoassociation with *L. plantarum* shortens adult life span relative to GF flies without accelerating ISC divisions. Instead, monoassociation with *L. plantarum* depletes ISC pools, blocks epithelial renewal, and damages the intestinal epithelium. A previous study showed that *Gluconobacter morbifer* causes disease in adult *Drosophila* if allowed to expand within the host (41). However, G. morbifer is a comparatively rare symbiont of *Drosophila*, and disease onset requires impaired immunity within the host. In contrast, this report identifies an intestinal phenotype associated with monoassociation of a common fly symbiont with a GF host. We believe that these findings represent a valuable model to define the mechanistic basis for symbiont-dependent stem cell damage.

At present, we do not know how monoassociation with *L. plantarum* causes an intestinal pathology within the host. It is possible that this phenotype arises from collateral damage through chronic expression of toxic immune effector molecules such as reactive oxygen species. This hypothesis is supported by the observation that *L. plantarum* activates NADPH-oxidase in the *Drosophila* intestine (32). Alternatively, errant intestinal immune responses through the immune deficiency (IMD) pathway may account for *L. plantarum*-dependent pathologies. In this context, we consider it important to consider that several transcriptional studies demonstrated that a relatively small fraction of IMD-responsive transcripts are easily categorized as bacteriostatic or immunomodulatory (42, 53). In fact, it seems that intestinal IMD activity primarily modifies metabolic gene expression (42, 43, 54). As intestinal microbes are known to control nutrition and metabolism in their *Drosophila* host (6, 51, 54, 55), we consider it possible that the *L. plantarum*-dependent pathologies described in this study reflect an underlying imbalance in IMD-dependent regulation of host metabolism. Consistent with possible links between *L. plantarum*, IMD, and host metabolism, it is noteworthy that a recent study established a link between *L. plantarum* and the IMD-dependent expression of intestinal peptidases (30). Our data show that intestinal colonization by *L. plantarum* is much greater in monoassociated flies than in polyassociated flies. We speculate that the elevated levels of *L. plantarum*, combined with the absence of additional symbionts, alter metabolic responses in the host, leading to impaired intestinal function. This hypothesis includes the possibility that *L. plantarum* directly affects host diet as proposed for other *Drosophila*-associated microbes (56–58).

Our work was initially inspired by reports from our group and others that GF adults outlive CR flies (9, 35). However, other studies reported variable impacts of the effects of microbiome removal on adult life span (59, 60). We believe that the differences between the individual reports reflect the intricate nature of interactions within a host-microbe-environment triad. For example, research groups typically raise their flies on an incompletely defined diet that exerts uncharacterized influences on the metabolic outputs of intestinal bacteria and the transcriptional outputs of the host. We believe that a complete evaluation of the relationship between microbes and their hosts requires consideration of environmental inputs such as diet.

A variety of host phenotypes have been associated with the presence and composition of *Lactobacillus* species associated with the gut of *Drosophila*. These phenotypes include effects on development (29), nutrition (57, 61), cell growth (32), immunity (33, 62), gene expression (42), and overall host fitness (47). Given the range of effects of lactobacilli on *Drosophila*, it is important to consider that individual species may be associated with multiple phenotypes in the host. For example, release of uracil from *L. brevis* promotes chronic generation of ROS that leads to an increase in intestinal apoptosis and decreased longevity (62), while *L. brevis* acts in association with *Acetobacter* to regulate triglyceride levels in the fly (61). Likewise, it is important to consider genotypic inputs from species strains associated with a given phenotype. For instance, the beneficial contributions of *L. plantarum* to mouse and larval nutrition display strain-specific effects (29, 31). Our study adds to this body of work through an examination of the impact of *L. plantarum* monoassociation with adult *Drosophila* on intestinal health and longevity.

In summary, this report uncovers long-term negative effects of Lactobacillus plantarum on the maintenance and growth of the intestinal stem cell pool. Given the experimental accessibility of *Drosophila* and *Lactobacillus*, we believe that these findings represent a valuable tool for the definition of the mechanisms by which individual symbionts influence stem cell homeostasis.

## MATERIALS AND METHODS

### Bacterial strains.

*Drosophila* symbiotic bacterial strains used were isolated from wild-type lab flies from the Foley lab at the University of Alberta and are as follows: Lactobacillus plantarum KP (DDBJ/EMBL/GenBank chromosome 1, GenBank accession number CP013749, and plasmids 1 to 3 for GenBank accession numbers CP013750, CP013751, and CP013752, respectively), Lactobacillus brevis EF (DDBJ/EMBL/GenBank accession number LPXV00000000), and Acetobacter pasteurianus AD (DDBJ/EMBL/GenBank accession number LPWU00000000). They are described in reference 37. Lactobacillus plantarum DF and Lactobacillus plantarum 39 have previously been described in references 37 and 48, respectively. *Lactobacillus* strains were grown in MRS broth (Sigma lot no. BCBS2861V) at 29°C, and Acetobacter pasteurianus was grown in mannitol broth (2.5% *n*-mannitol, 0.5% yeast extract, 0.3% peptone) at 29°C with shaking.

### CFU per fly.

At indicated time points, 25 flies were collected from an indicated group and placed into successive solutions of 20% bleach, distilled water, 70% ethanol, and distilled water to surface sterilize and rinse flies, respectively. These 25 flies were then randomly divided into groups of 5 and mechanically homogenized in MRS broth. Fly homogenate was then diluted in serial dilutions in a 96-well plate, and 10-µl spots were plated on either MRS agar to select for *Lactobacillus* species or GYC agar to select for *Acetobacter*. Plates were incubated for 2 days at 29°C, and the number of colonies per bacterial species was counted. *L. plantarum* colonies were identified on MRS agar as round, solid white, opaque colonies that grew to easily visible colonies at 29°C in 2 days. *L. brevis* colonies were identified as large, round, irregular-edged colonies on MRS agar with an off-white center fading to translucence at the edges of the colony that grew to easily visible colonies at 29°C in 2 days. *A. pasteurianus* colonies were identified on GYC agar as small, round, beige, translucent colonies that grew to visually identifiable colonies at 29°C in 3 days and began to clear calcium carbonate from the GYC plate in 4 days. To distinguish between bacterial species in coassociations and those in polyassociations, bacterial colony morphology was scrutinized under a dissecting microscope.

### Fly husbandry.

All experiments were performed with virgin female flies. *w*^*1118*^ flies were used as the wild-type strain and used in all experiments unless otherwise mentioned. Flies were raised on standard cornmeal medium (Nutri-Fly Bloomington formulation; Genesee Scientific) at 29°C. The *w, esg-GAL4, tubGAL80*^*ts*^*, UAS-GFP* flies have previously been described (45, 63). Mitotic clones were generated with flies of the genotype *y,w, hs-flp, UAS-mCD8GFP; neoFRT(40A)/neoFRT(40A), tubGAL80; tubGAL4/*^*+*^. Germfree flies generated by antibiotic treatment were made by raising freshly eclosed adult flies on autoclaved standard medium supplemented with an antibiotic solution (100 µg/ml ampicillin, 100 µg/ml metronidazole, 50 µg/ml vancomycin dissolved in 50% ethanol, and 100 µg/ml neomycin dissolved in water) to eliminate the microbiome from adult flies (41). Throughout this study, we confirmed microbial elimination from adult flies at various points during experiments by plating whole-fly homogenates on agar plates permissive for the growth of *Lactobacillus* and *Acetobacter*. CR flies were raised on autoclaved standard cornmeal medium. To obtain axenic fly stocks from embryo, embryos were laid on apple juice plates over a 16-h period and then collected. All the following steps were performed in a sterile hood. Embryos were rinsed from the plate with sterile phosphate-buffered saline (PBS). Embryos were placed in 10% sodium hypochlorite solution for 2.5 min, then placed into fresh 10% sodium hypochlorite solution for 2.5 min, and then washed with 70% ethanol (EtOH) for 1 min. Embryos were then rinsed 3 times with sterile water, placed onto sterile food, and maintained at 25°C in a sterilized incubator in a sterile hood. Axenic flies were generated in parallel with conventionally reared counterparts who were placed in water at all steps. For longevity studies, 100 flies were raised in vials with 20 flies per vial. Flies were passed to fresh food every Monday, Wednesday, and Friday, and dead flies were counted at each passage.

### Generation of gnotobiotic *Drosophila*.

Virgin females were raised on antibiotic-supplemented medium for 5 days at 29°C. On day 5 of antibiotic treatment, a fly from each group was homogenized in MRS broth and plated on MRS and GYC agar plates to ensure eradication of preexisting microbes. Flies were starved in sterile empty vials for 2 h prior to bacterial association. For monoassociations, the optical density at 600 nm (OD_600_) of bacterial liquid cultures was measured and then the culture was spun down and resuspended in 5% sucrose in PBS to a final OD_600_ of 50. For axenic embryos, virgin female flies were collected for 2 to 3 days and then associated with the same protocol as antibiotic-treated GF flies. For coassociations, bacterial cultures of *A. pasteurianus* and *L. plantarum* were prepared to an OD_600_ of 50 in 5% sucrose in PBS as described above. The bacterial cultures were then mixed at ratios of 1,000:1, 100:1, 10:1, and 1:1 *A. pasteurianus* to *L. plantarum* bacteria. For polyassociations, bacterial cultures of *A. pasteurianus*, *L. brevis*, and *L. plantarum* were prepared to an OD_600_ of 50 in 5% sucrose in PBS as described above. The bacterial cultures were then mixed at a 1:1:1 ratio. For all bacterial associations, 22 flies/vial were associated with 1 ml of bacterial suspension on autoclaved cotton plugs. Flies were fed a bacterium-sucrose mixture for 16 h at 29°C and then kept on autoclaved food for the remainder of the study. CR and GF flies were given mock associations of 1 ml of 5% sucrose in PBS for 16 h at 29°C. To ensure monoassociation or GF conditions, sample flies were homogenized in MRS broth and plated on MRS or GYC agar plates periodically throughout the study.

### Immunofluorescence.

Flies were washed with 95% ethanol and dissected in PBS to isolate adult intestines. Guts were fixed for 20 min at room temperature in 5% formaldehyde in PBS. Guts were rinsed in PBS for 20 min at room temperature and blocked overnight in PBSTBN (PBS, 0.05% Tween 20, 5% bovine serum albumin [BSA], and 1% goat serum) at 4°C. Guts were stained overnight at 4°C in PBSTBN with appropriate antibodies, washed with PBSTB (PBS, 0.05% Tween 20, and 5% BSA) and stained for 1 h at room temperature in PBSTBN with Hoechst 33258 (1:500 from Molecular Probes Life Technologies) and the appropriate secondary antibody (1:500 goat anti-mouse Alexa Fluor 568 or 1:500 goat anti-mouse Alexa Fluor 647 from Invitrogen Molecular Probes). Guts were washed with PBSTB and rinsed with PBS prior to visualization. The primary antibodies used in this study were as follows: mouse anti-Armadillo (1:100; Developmental Studies Hybridoma Bank N2 7A1) and mouse anti-Prospero (1:100; Developmental Studies Hybridoma Bank MR1A). Guts were mounted on slides in Fluoromount (Sigma-Aldrich F4680), and the posterior midgut was visualized with a spinning disk confocal microscope (Quorum WaveFX; Quorum Technologies Inc.). Images were collected as z-slices and processed with Fiji software to generate a single z-stacked image.

### qPCR.

Real-time PCR was performed on the dissected guts of adult *Drosophila*. The quantitative PCR (qPCR) protocol and primers used in this study have been described previously (64).

### Quantification of gut length.

The guts of aged flies were dissected and immediately mounted on slides. Length was measured with an eyepiece micrometer (Motic B1-220 series system microscopes) at ×4 magnification (Motic 4/0.10 160/0.17 lens) by tracing from posterior of the proventriculus along the midgut to just anterior of the midgut-hindgut junction (identified by the branching of the Malpighian tubules).

### Statistical analysis.

Significant differences in longevity were determined by a log rank (Mantel-Cox) test using GraphPad Prism 6.0, followed by pairwise comparisons between treatment groups. To identify significant changes in gene expression, clones/sample, cells/clone, and GFP-positive cells/area, samples were tested with analysis of variance (ANOVA) with Bonferroni corrections for multiple samples using GraphPad Prism 6.0.

### Microarray data comparison.

Comparisons were performed on genes previously characterized as microbe responsive in the intestines of adult *Drosophila* (42, 43). For this study, we defined genes with greater than 1.5-fold expression changes as differentially regulated. We then used PANTHER (65) to identify gene ontology terms with a minimum of five genes that were enriched in the respective groups. Metadata are available in Data Set S1 in the supplemental material.

10.1128/mBio.01114-18.1 DATA SET S1  Differentially regulated microbe-dependent gene expression. Differentially regulated genes in the intestines of Oregon R or Canton S flies raised from axenic embryos relative to the intestines of CR Oregon R or Canton S flies, respectively (42). Differentially regulated genes in the intestines of antibiotic-treated *esg*^*ts*^ flies relative to the intestines of CR *esg*^*ts*^ flies (43). Download DATA SET S1, XLSX file, 0.8 MB. Copyright © 2018 Fast et al. 2018 Fast et al. This content is distributed under the terms of the Creative Commons Attribution 4.0 International license.

### Transmission electron microscopy.

Flies were washed with 95% ethanol and dissected into PBS. Posterior midguts were immediately excised and placed into fixative (3% paraformaldehyde plus 3% glutaraldehyde). Fixation preparation, contrasting sectioning, sectioning, and visualization were performed at the Faculty of Medicine and Dentistry Imaging Core at the University of Alberta. The midgut sections of 2 separate flies per treatment were visualized with a Hitachi H-7650 transmission electron microscope at 60 kV in high-contrast mode.
